# Early Diagnosis of Ovarian Cancer: A Comprehensive Review of the Advances, Challenges, and Future Directions

**DOI:** 10.3390/diagnostics15040406

**Published:** 2025-02-07

**Authors:** Mun-Kun Hong, Dah-Ching Ding

**Affiliations:** 1 Department of Obstetrics and Gynecology, Hualien Tzu Chi Hospital, Buddhist Tzu Chi Medical Foundation, Tzu Chi University, Hualien 970, Taiwan; jeff06038@gmail.com; 2 Institute of Medical Sciences, Tzu Chi University, Hualien 970, Taiwan

**Keywords:** ovarian cancer, pathogenesis, early diagnosis, multiomics, tumor markers

## Abstract

Ovarian cancer (OC), the seventh most common cancer in women and the most lethal gynecological malignancy, is a significant global health challenge, with >324,000 new cases and >200,000 deaths being reported annually. OC is characterized by late-stage diagnosis, a poor prognosis, and 5-year survival rates ranging from 93% (early stage) to 20% (advanced stage). Despite advances in genomics and proteomics, effective early-stage diagnostic tools and population-wide screening strategies remain elusive, contributing to high mortality rates. The complex pathogenesis of OC involves diverse histological subtypes and genetic predispositions, including *BRCA1/2* mutations; notably, a considerable proportion of OC cases have a hereditary component. Current diagnostic modalities, including imaging techniques (transvaginal ultrasound, computed/positron emission tomography, and magnetic resonance imaging) and biomarkers (CA-125 and human epididymis protein 4), with varying degrees of sensitivity and specificity, have limited efficacy in detecting early-stage OC. Emerging technologies, such as liquid biopsy, multiomics, and artificial intelligence (AI)-assisted diagnostics, may enhance early detection. Liquid biopsies using circulating tumor DNA and microRNAs are popular minimally invasive diagnostic tools. Integrated multiomics has advanced biomarker discovery. AI algorithms have improved imaging interpretation and risk prediction. Novel screening methods including organoids and multiplex panels are being explored to overcome current diagnostic limitations. This review highlights the critical need for continued research and innovation to enhance early diagnosis, reduce mortality, and improve patient outcomes in OC and posits personalized medicine, integrated emerging technologies, and targeted global initiatives and collaborative efforts, which address care access disparities and promote cost-effective, scalable screening strategies, as potential tools to combat OC.

## 1. Introduction

Ovarian cancer (OC) is a complex disease; 324,603 new OC cases were reported in 2022 [1]. According to the World Health Organization (WHO), >200,000 women die from OC annually worldwide [2]. A lethal gynecological malignancy, OC is the seventh most common cancer in women globally [3]. It is a heterogeneous disease with various subtypes, primarily of epithelial-, stromal-, or germ-cell origin [4]. Recent evidence suggests a fallopian tube origin, rather than an ovarian origin, for the most prevalent subtype, high-grade serous ovarian cancer (HGSOC) [5]. The high mortality rate of OC is partly attributed to late-stage diagnosis and the cancer’s unique metastatic process, which involves a leader cell-driven collective invasion [4]. Despite advances in genomics and proteomics, progress in clinical management has been limited [6]. Current research is focused on understanding the molecular processes involved in OC development, exploring potential chemotherapeutics, and investigating factors that drive the initiation and migration of dysplastic cells from the fallopian tube to the ovary [5,6,7].

Approximately 66% of patients are diagnosed at advanced stages, International Federation of Gynecology and Obstetrics or “FIGO” stage III or IV, which have a 5-year survival rate of 41% and 20%, respectively [8]. Conversely, the 5-year survival rates for stages I and II are approximately 93% and 74%, respectively [8]. Therefore, early-stage detection and treatment of OC are vital.

However, OC often presents with vague and non-specific symptoms, challenging its early detection [9]. The main symptoms associated with advanced-stage OC are bloating (77%), increased abdominal size (64%), abdominal pain (22%), constipation (24%), back pain (45%), pelvic pain (26%), fatigue (34%), and urinary urgency or frequency (16–34%) [10]. Nevertheless, the positive predictive value (PPV) of OC symptoms alone remains relatively low, approximately 0.6–1.1% overall [11,12]. Most early-stage OCs are asymptomatic, significantly compounding the challenge of timely detection [13].

This review focuses on the early diagnosis of OC, comprehensively summarizing the disease’s pathophysiology and risk factors, current diagnostic approaches, screening strategies, emerging technologies, and the associated challenges.

## 2. Search Strategy

A systematic search was conducted using the keywords “ovarian cancer” and “diagnosis”, including their synonyms and related terms, in the PubMed, Scopus, Web of Science, and Embase databases. The search covered studies from the inception of each database to 24 December 2024. Additionally, the reference lists of relevant reviews and selected studies were examined. Table 1 outlines the detailed search strategy.

## 3. Pathophysiology and Risk Factors

### 3.1. Pathogenesis and Histological Subtypes of OC

OC has multiple histological subtypes, each with its own unique pathogenesis and clinical implications. OC pathogenesis has evolved through several models, and the current model focuses on tumor origins and their genetic underpinnings.

#### 3.1.1. Epithelial Tumors (Approximately 90% of Cases)

##### High-Grade Serous Carcinoma (HGSC)

HGSC is the most common and lethal subtype of OC, accounting for significant morbidity and mortality [14,15]. It is often associated with *BRCA1* and *BRCA2* mutations [16]. Although HGSCs were believed to originate from the ovarian surface epithelium (OSE), recent evidence suggests their origin from the fallopian tube, with serous tubal intraepithelial carcinoma (STIC) as a possible precursor lesion [16,17]. *TP53* mutations and genotoxic stress (ovulatory cytokines + reactive oxygen species) in the fallopian tube lead to the formation of STICs, which can progress to invasive cancer [7,18]. Evidence indicating that p53 signatures are more common in the fallopian tubes than in the OSE or cortical inclusion cysts (CICs) supports this theory [19]. The precise cellular origin and molecular characteristics of HGSC need elucidation to improve early detection, prevention, and treatment strategies [16,17].

##### Low-Grade Serous Carcinoma (LGSC)

LGSC of the ovary is a rare subtype of epithelial OC that typically arises from benign precursor CICs. It is characterized by a young age at diagnosis, indolent course, and prolonged survival compared to HGSC [20,21]. LGSC typically presents with high *KRAS* and *BRAF* mutations and low *TP53* mutations [21]. Treatment primarily involves surgery and adjuvant platinum-based chemotherapy as standard care, despite LGSC being relatively chemoresistant [20,22]. Hormonal therapy, particularly post-chemotherapy maintenance therapy, has demonstrated benefits [20,23]. With the identification of potential therapeutic targets, including the MAP kinase, IGF-1R, and angiogenesis pathways, MEK inhibitors, *BRAF* inhibitors, and bevacizumab hold promise as effective treatments [23]. The unique molecular profile and clinical behavior of LGSCs indicate the need for specialized treatments, including targeted therapies, and further investigations [20,23].

##### Endometrioid Carcinoma (EOC)

Often associated with endometriosis, EOCs comprise 5–10% of all OCs [24,25]. EOCs frequently harbor *KRAS*, *PIK3CA*, *PTEN*, *CTNNB1*, *ARID1A*, and *TP53* mutations, distinguishing them from HGSCs [25]. EOC is classified into four molecular subtypes: POLE (ultramutated), MSI (hypermutated), high copy number (serous-like), and low cop number (endometrioid) [25]. EOCs are less likely to have nodal metastases, and *BRCA1/2* mutations are less common in EOCs than in HGSCs [26]. The presence of endometriosis in pathological sections decreases with an advancing stage [24]. EOCs are often associated with concurrent endometrial cancer and have distinct clinicopathological characteristics [24]. Understanding these molecular subtypes and characteristics may aid prognosis prediction and targeted therapy development [27].

##### Clear Cell Carcinoma of the Ovary (CCCO)

CCCO is a rare subtype of epithelial OC with distinct clinical and genetic features [28]. This rare, yet aggressive, tumor is more prevalent in Asian populations and is often associated with endometriosis [29,30]. CCCO is typically diagnosed at earlier stages and in younger women than other OCs [30]. Although an early-stage prognosis is favorable, advanced or recurrent disease has poor outcomes owing to chemoresistance [28,30]. Common genetic alterations include *ARID1A* and *PIK3CA* mutations [30]. Treatment for advanced CCCO involves cytoreductive surgery and platinum-based chemotherapy, similar to that for HGSC [30]. However, targeted therapies, such as PI3K/AKT/mTOR pathway inhibitors, hold promise as future treatments [31]. CCCO’s rarity necessitates international collaboration for conducting clinical trials and improving patient outcomes [28,31].

##### Mucinous Ovarian Carcinoma (MOC)

MOC is a rare subtype of epithelial OC with distinct clinical and molecular characteristics [32,33]. It is characterized by mucin-rich cystic cavities and often presents as a large, unilateral adnexal mass [33,34]. Early-stage MOC has an excellent prognosis, with a survival rate of >90% for stage IA [33]. However, advanced MOC responds poorly to conventional platinum-based chemotherapy and PARP inhibitors [34]. Accurate diagnosis is crucial, as distinguishing primary from metastatic MOCs can be challenging [32,35]. Mucins influence MOC development and may aid differential diagnosis and targeted therapies [34]. Recent molecular insights may offer improved clinical management and treatment strategies [35].

#### 3.1.2. Sex Cord-Stromal Tumor (SCST) (11.4% of Cases)

SCSTs are rare neoplasms, accounting for approximately 7% of primary ovarian tumors [36]. They arise from ovarian connective tissue, namely stromal cells and primitive sex cords, and encompass various subtypes with distinct histological features and biological behaviors [37]. Typically presenting at an early stage, SCSTs have a good prognosis; however, they may recur up to 30 years after initial treatment [38]. The primary treatment is surgery, with no evidence supporting adjuvant therapy for stage IA or IB tumors [38]. Platinum-based chemotherapy is used for advanced or recurrent disease [38]. SCSTs often produce hormones, leading to various endocrine syndromes [36]. Imaging techniques such as computed tomography (CT) and magnetic resonance imaging (MRI) can help differentiate SCSTs from more common epithelial tumors because of each subtype’s characteristic features [39]. The tumor’s indolent nature necessitates long-term follow-up [38].

#### 3.1.3. Germ Cell Tumor (GCT) (Approximately 10% of Cases)

Ovarian GCTs are a rare, diverse group of neoplasms of primitive germ-cell origin, accounting for 15–20% of all ovarian tumors [40]. While most are benign mature cystic teratomas, malignant ovarian GCTs comprise approximately 5% of GCTs and 2.6% of all OCs [40,41]. Malignant ovarian GCTs typically affect young women and are characterized by abdominal pain, palpable mass, and elevated tumor markers [41]. Common types include dysgerminoma, immature teratoma, and yolk sac tumors, each with distinct imaging features [41]. Overlapping morphology with other tumors complicates the diagnosis, necessitating immunohistochemical staining [42]. Treatment involves surgery and chemotherapy, with the bleomycin, etoposide, and cisplatin protocol being crucial [43]. While recent advances have improved the prognosis and enabled fertility-conserving surgeries, research on novel therapeutic approaches is ongoing [43].

### 3.2. Genetic Predispositions

A genetic predisposition contributes considerably to OC development; notably, approximately 23% of cases have a hereditary component [44]. *BRCA1* and *BRCA2* mutations account for 20–25% of HGSCs [44]. Women with *BRCA1* and *BRCA2* mutations have a 39% and 11% risk, respectively, of developing OC by the age of 70 [45]. Other genes associated with hereditary OCs include *TP53*, *PTEN*, *STK11*, *CDH1*, *PALB2*, *BRIP1*, *ATM*, *CHEK2*, and mismatch repair genes [46]. Genetic testing is crucial for identifying at-risk individuals, guiding prevention strategies and informing treatment decisions, such as PARP inhibitor therapy [45]. Current international guidelines recommend *BRCA1/2* mutation testing for all patients with OC, regardless of age or family history [45,47].

### 3.3. Lifestyle and Environmental Risk Factors

OC is a lethal gynecological malignancy with various risk factors. Tall stature, high body mass index, and hormone replacement therapy have an increased OC risk, while oral contraceptive use has a decreased risk [48]. Mendelian randomization studies have confirmed these findings and identified additional risk factors, including early menarche and endometriosis [49]. Regarding environmental factors, a potential link between water pollutants from pulp and paper mills and OC incidence has been reported [50]. Recognized protective factors include parity and oral contraceptive use, while non-modifiable factors include family history of ovarian and certain other cancers [51]. However, recognized risk factors explain only a limited proportion of cases. The recent favorable trends in OC incidence and mortality in high-income countries have largely been attributed to widespread oral contraceptive use among young women [51].

### 3.4. High-Risk Populations and Implications for Screening

OC screening and prevention remain challenging, particularly for high-risk populations. Risk-reducing salpingo-oophorectomy is the most effective prevention strategy for high-risk women, despite its notable side effects [52]. Opportunistic bilateral salpingectomy is being explored for the general population [53,54]. While population-based screening has not demonstrated mortality benefits, multimodal screening using longitudinal CA-125 algorithms may help detect early-stage disease [53]. However, current screening methods lack sensitivity and specificity for early-stage detection, especially in the general population [55]. Strategies such as more frequent multimodal screening and chemoprevention with oral contraceptives are being investigated for high-risk women, particularly those with *BRCA1/2* mutations [53]. Future research should focus on developing novel biomarkers, improving risk prediction models, and evaluating changing exposure patterns in diverse populations [56].

## 4. Current Diagnostic Approaches

### 4.1. Imaging Techniques

#### 4.1.1. Role of Transvaginal Ultrasound (TVUS)

TVUS is a promising tool for diagnosing OC, despite its variable diagnostic accuracy. The Ovarian-Adnexal Reporting and Data System (O-RADS) demonstrated a sensitivity and specificity of 52% and 84%, respectively, for detecting malignant ovarian neoplasms [57]. A large-scale trial reported that TVUS alone had a sensitivity and specificity of 84.9% and 98.2%, respectively, while a combination of CA-125 and TVUS had an improved sensitivity and specificity of 89.4% and 99.8%, respectively [58]. Another study reported a specificity and PPV of 98.5% and 8.9%, respectively, for TVUS [59]. Notably, a prospective evaluation of TVUS in detecting pelvic carcinomatosis in patients with OC reported a sensitivity of 84%, specificity of 96%, and overall accuracy of 89% [60]. These findings suggest that TVUS, especially in combination with other modalities, is a valuable OC diagnostic tool.

#### 4.1.2. Advances in Imaging Modalities and Diagnostic Accuracy

Imaging modalities, including ultrasound, CT, and MRI, essentially complement biomarkers [61]. All three modalities demonstrate a high overall accuracy for malignancy diagnosis [62].

CTs demonstrate limited diagnostic performance in detecting lymph node metastases and residual disease in patients with OC. The sensitivity and specificity of CT are 40.7–92.16% and 57.14–89.1%, respectively [63,64]. CT and laparoscopy have a comparable accuracy in predicting the peritoneal cancer index (PCI), with a sensitivity of 94.9% and 98.3%, respectively [65]. However, CT has a lower negative predictive value (NPV) than laparoscopy, especially for non-measurable lesions and specific anatomical sites [64]. The combination of CT and exploratory laparoscopy significantly improves the diagnostic power in detecting bowel involvement, increasing the sensitivity from 56.7% to 87.5% [66]. While CT alone may be insufficient for surgical planning, a standardized CT-PCI and laparoscopy can enhance the assessment of the disease extent and guide treatment decisions in patients with OC [65,66].

MRI has an accuracy that is superior to Doppler ultrasound and CT in diagnosing malignant ovarian masses [62]. Diffusion-weighted MRI offers a high contrast between tumor and healthy tissue, aiding in disease staging and response assessment [67]. The superior soft-tissue contrast of MRI allows for accurate differentiation between benign and malignant adnexal masses, as well as borderline tumors [68]. Deep learning models based on convolutional neural networks have recently been reported to achieve a diagnostic performance comparable to that of experienced radiologists in identifying ovarian carcinomas on MRI [69]. The O-RADS MRI risk score was developed to standardize cancer risk scoring, potentially reducing unnecessary interventions, while expediting care for patients with OC [70]. MRI has been reported to have a sensitivity and specificity of 98% and 83%, respectively, in detecting ovarian tumors [71]. Moreover, MRI has proven effective in diagnosing ovarian endometriosis, with a sensitivity and specificity of 86.7% and 81.9%, respectively [72]. Furthermore, gadolinium-enhanced MRI outperformed CA-125 and physical examinations in detecting residual tumors in patients treated for OC, with a sensitivity and specificity of 91% and 87%, respectively [73]. Nevertheless, other diagnostic tools, such as the Risk of Malignancy Index, which combines ultrasound findings, menopausal status, and serum CA-125 levels, have demonstrated high sensitivity (89.5%) and specificity (96.2%) in identifying malignant ovarian tumors as well [74].

Integrated molecular imaging techniques, particularly [^18^F]fluorodeoxyglucose (FDG)-positron emission tomography (PET)/CT, can potentially improve staging and treatment planning [61]. PET/CT has revolutionized OC management, impacting staging, treatment planning, and recurrence detection [75]. FDG PET/CT is reportedly more effective than conventional imaging in detecting disease progression (37.93% vs. 17.24%) and recurrence (24.14% vs. 6.90%) in patients with OC [76]. For recurrent OC detection, FDG-PET demonstrated a high sensitivity (84.6–90%) and specificity (100%) [77]. However, its performance in lymph node assessment varied, with higher specificity (90.9%) albeit lower sensitivity (26.7%) [78]. For diagnosis of the peritoneal carcinomatosis extent, with an accuracy of 41.7–67.8% depending on the lesion site, PET/CT performs less effectively than standard CT [79]. Despite some limitations, PET/CT significantly influences treatment strategies, leading to therapeutic changes in 55.17% of patients compared with 17.24% for conventional imaging [76]. Ongoing research is focused non-FDG tracers, such as fibroblast activation protein inhibitors, for potential theragnostic applications [75].

Advancements notwithstanding, challenges persist in achieving ideal sensitivity and specificity for early detection of OC [80] (Table 2).

### 4.2. Biomarkers

#### 4.2.1. CA-125 and Its Limitations

Serum CA-125 has long been the primary biomarker for OC detection; however, its application is limited in early-stage diagnosis and population-based screening [81]. Its diagnostic sensitivity is low for early-stage disease and can be high for non-cancerous conditions [82]. Combined CA-125 and human epididymis secretory protein 4 (HE4) assays have demonstrated an improved diagnostic efficiency, with an area under the curve (AUC) of up to 0.96 [82]. However, recent research suggests that even this combination is insufficient to detect early-stage disease [83]. Multivariate index assays incorporating CA-125, HE4, and patient characteristics can potentially improve specificity and sensitivity in early OC detection [84]. Other biomarker combinations, such as OVA1, Risk of Ovarian Malignancy Algorithm (ROMA), and Overa, demonstrate potential as well. Nevertheless, significant challenges remain in developing a reliable screening method for early-stage OC [83,84].

The combination of CA-125 and a TVUS-based tumor-morphology index (MI) is effective in identifying ovarian tumors at a high risk of malignancy. While one study reported that an MI score ≥ 5 correlated with a significant risk of malignancy, another reported a sensitivity and specificity of 98.1% and 80.8%, respectively, for predicting malignancy at an MI threshold of 5 [85,86].

#### 4.2.2. PPV and NPV of CA-125

The Risk of Ovarian Cancer Algorithm (ROCA), which evaluates serum CA-125 levels over time, is a potential tool for early detection, with a high specificity (92%) and an improved early-stage sensitivity compared with standard CA-125 cutoffs [87]. One study found that CA-125 had moderate sensitivity (80.1%) and specificity (53.6%), with a low PPV of 48.4% and a high NPV of 83% [88]. Another study reported a specificity and PPV of 99.9% and 40%, respectively, for ROCA followed by TVUS [89]. The combination of symptoms with CA-125 testing demonstrated a sensitivity and specificity of 89.3% and 83.5%, respectively, in detecting OC [90]. These findings suggest that while CA-125 alone may not be ideal for screening, its use in algorithms, such as ROCA, and in combination with other methods can improve early detection of OC.

#### 4.2.3. Emerging Biomarkers

Recent research has highlighted the potential of emerging biomarkers (e.g., HE4 and OVA1) for improving OC diagnosis. The efficacy of the widely used CA-125 is limited by its low specificity [91]. HE4 is a promising complementary biomarker, with a performance superior to that of CA-125 in predicting tumor malignancy and recurrence [92]. Multiplex panels combining CA-125, HE4, and other tools, such as the ROMA and OVA1, have been developed to enhance diagnostic accuracy [84,91]. The triple screen assay (CA-125, HE4, and symptom index) demonstrated a sensitivity of 79%, specificity of 91%, PPV of 83%, and NPV of 89% [93]. These approaches have demonstrated improved sensitivity and specificity over single-marker tests. Ongoing research is focused on novel biomarkers such as autoantibodies, circulating tumor DNAs (ctDNA), miRNAs, and DNA methylation signatures [91]. Additionally, the potentiality of aptamers as a tool for identifying tumor-specific antigens for early diagnosis and targeted therapy is being investigated [94] (Table 3).

#### 4.2.4. Multiplex Panels for Improved Specificity and Sensitivity

Recent studies have investigated multiplex panels for improving OC diagnosis. A microfluidic platform demonstrated high specificity and low cross-reactivity for a four-marker panel (CA-125, HE4, MMP-7, and CA72-4), distinguishing cases from controls with a sensitivity and specificity of 68.7% and 80%, respectively [95]. A combination of CA-125 with transthyretin and apolipoprotein A1 reportedly achieved a sensitivity and specificity of 95% and 97%, respectively, significantly improving early-stage detection [96]. A multiplex methylation-specific polymerase chain reaction (PCR) assay examining seven genes in ctDNA demonstrated a sensitivity and specificity of 85.3% and 90.5%, respectively, for stage I disease, outperforming CA-125 used alone [97]. Using a novel multiplex platform, researchers identified 38 significant protein biomarkers and demonstrated an enhanced sensitivity of 93–95% and a specificity of 95% for a 12-protein multiplex panel [98]. These studies demonstrate the potential of multiplex panels to improve OC diagnosis, particularly early-stage detection.

### 4.3. Molecular and Genetic Tests

#### 4.3.1. Liquid Biopsy and ctDNA

Liquid biopsy, which uses circulating tumor cells (CTCs) and ctDNA, is a minimally invasive approach for OC diagnosis, prognosis, and treatment monitoring [99,100]. These biomarkers correlate with the tumor burden and enable comprehensive molecular profiling of primary, metastatic, and recurrent tumors [99]. Recent studies on the clinical potential of CTCs and ctDNA in OC management have demonstrated their value in early detection, prognosis assessment, and treatment response evaluation [101,102]. Additionally, cell-free microRNAs and exosomes are effective liquid biopsy tools for OC [101,102]. Although liquid biopsy may help improve OC outcomes, further research is needed to address certain challenges before its implementation in routine clinical practice [99,100].

#### 4.3.2. Role of Genomics in Early Detection

Genomic approaches for early diagnosis of OC have been extensively explored. Next-generation sequencing (NGS) has identified novel somatic mutations and copy number alterations in patients with OC, providing potential markers for early detection [103]. Despite their similar mutation profiles, late-stage HGSC exhibits higher ploidy and genomic instability than early-stage HGSC [104]. NGS panels can identify actionable genetic alterations, potentially guiding targeted therapies and genetic counseling [105]. RNA sequencing offers advantages over conventional methods, providing deeper insights into gene expression, alternative splicing, and novel transcripts in OC [106]. A study on NGS-based genomic profiling revealed novel mutations in Chinese patients with OC, furthering our understanding of the disease’s molecular mechanisms [103]. NGS has identified novel somatic mutations in patients with OC, with 463 potential pathogenic sites assigned to 473 genes [103].

Cell-free DNA (cfDNA) analysis is a promising non-invasive diagnostic tool for OC. A multiomics approach combining copy number variation, 5′-end motifs, fragmentation profiles, and nucleosome footprinting reportedly achieved high accuracy, up to 91%, in distinguishing patients with OC from healthy controls [107]. Additionally, cfDNA methylation profiling has demonstrated potential for early OC detection, with a diagnostic accuracy of up to 91% [108]. Thus, genomic approaches offer new possibilities for improving early diagnosis and understanding the molecular mechanisms of OC development.

#### 4.3.3. Role of Proteomics in Early Detection

Recent advances in genomics and proteomics hold much promise for early OC detection [109]. Mass spectrometry-based proteomics techniques have emerged as powerful tools for biomarker discovery and characterization of molecular pathways in OC [110,111]. These approaches offer improved sensitivity and specificity compared with conventional diagnostic methods such as CA-125 and HE4 assays [110]. Integrated multiomics, combining genomic, transcriptomic, proteomic, and metabolomic data, has augmented our understanding of OC and identified potential novel biomarkers [109]. Furthermore, proteomics analysis can uncover new therapeutic targets and help predict drug resistance, potentially improving patient outcomes [112]. Nevertheless, challenges remain owing to the complexity and heterogeneity of OC, as well as limitations in mass spectrometry techniques [110].

## 5. Screening Strategies

Current OC screening guidelines emphasize the importance of targeted screening for high-risk women. For the general population, the United States Preventive Services Task Force recommends against OC screening for asymptomatic women who are not at high risk [113]. This recommendation is based on evidence indicating that such screening does not reduce mortality and can lead to considerable harm, including false-positive results and unnecessary surgeries [55,114].

Regarding screening tests, commonly used tests, such as TVUS and the serum CA-125 test, have not effectively reduced OC mortality among average-risk women [115]. Therefore, major medical organizations do not recommend these tests for routine screening in this group.

For high-risk populations, i.e., women with *BRCA1/2* mutations or those with a family history of hereditary syndromes such as the Lynch syndrome, are advised to undergo regular screenings, including a combination of TVUS and serum CA-125 tests, biannually, typically starting at the age of 30, especially for those with *BRCA1/2* mutations [55,114]. Women with *BRCA* mutations may consider prophylactic surgery (salpingo-oophorectomy) as well to reduce their risk of developing OC [116]. Moreover, recent studies indicate that salpingectomy is an effective strategy for reducing the OC risk in the general population. Prophylactic bilateral salpingectomy should be considered for women undergoing hysterectomy for benign conditions or those seeking sterilization [54,117].

## 6. Emerging Technologies

### 6.1. Artificial Intelligence (AI) and Machine Learning

#### 6.1.1. Use of AI for Pattern Recognition in Imaging and Biomarkers

AI has the potential to enhance OC diagnosis and management. AI techniques, including machine and deep learning, have been applied to CT, MRI, and ultrasound for cancer detection and classification [118]. A meta-analysis revealed the favorable diagnostic performance of AI algorithms, with a pooled sensitivity and specificity of 88% and 85%, respectively [119]. AI-based radiomics is a non-invasive and economical approach for OC assessment [120]. However, challenges persist, including limited data availability owing to low disease prevalence and the need for publicly accessible imaging datasets [121]. AI’s potential in improving diagnostic and prognostic capabilities notwithstanding, most models are yet to be applied in clinical settings, and regulatory approval for AI-based imaging biomarkers remains pending [121]. Continued efforts to develop explainable and trustworthy AI models are necessary for effective biomarker discovery in rare cancers.

#### 6.1.2. AI-Assisted Risk Prediction Models

AI application in OC diagnosis and risk prediction have been the focus of recent research. AI models have demonstrably been effective in predicting OC from preoperative examinations, with a diagnostic accuracy of 80–86% [122,123]. Machine learning algorithms, including XGBoost, Random Forest, and Support Vector Machine, have been used to develop these predictive models [124,125]. The key predictive factors identified include tumor markers such as HE4 and CA-125, as well as blood test results [123]. AI-assisted models can potentially aid in early, easy, and less expensive OC diagnosis [123]. Nevertheless, future research should focus on integrating imaging data with serum biomarkers to further improve diagnostic accuracy [123,124].

### 6.2. Novel Diagnostic Tools

#### 6.2.1. Organoids and Their Potential in Early Detection

Organoids are emerging tools for OC research, offering advantages over conventional cell lines and xenografts [126,127]. These three-dimensional cultures accurately mimic tumor phenotypes, enabling studies on cancer heterogeneity and drug screening [126,128]. Organoids derived from murine, healthy individuals, and patient-origin tissues replicate the morphological, histological, and genetic attributes of OC [129]. They serve as preclinical models for predicting treatment responses and guiding personalized therapies [129]. Additionally, organoids facilitate the investigation of cancer progression, metastasis, and drug resistance mechanisms [129]. In HGSOC, organoids have been used to assess cells of origin and perform drug sensitivity testing [130,131]. Current organoids are limited to epithelial cells; however, future models may incorporate microenvironments for cell–cell and cell–matrix interactions [126], potentially revolutionizing OC research and personalized medicine strategies.

#### 6.2.2. Role of Wearable Technologies and Remote Monitoring

Wearable technologies and remote monitoring are valuable tools in OC care. These devices can continuously collect real-time data on patients’ health status, reflecting changes in functional status, symptom burden, and quality of life [132]. Electronic patient-reported outcome systems for monitoring patients with OC for relapse reportedly have high compliance rates and patient satisfaction [133]. Wearable smart systems can track various health parameters, offering cost-effective solutions for remote clinical trial monitoring in cancer research [134]. These technologies may enable more proactive and personalized care, although challenges in data management, patient engagement, and integration into existing healthcare systems remain [132]. Additionally, studies on novel bioengineering advances, such as liquid biopsies, for improving surveillance and treatment outcomes for patients with epithelial OC are ongoing [135].

### 6.3. Methylation Profile of Cervical Scrapings

Recent studies have investigated the potentiality of DNA methylation in cervical scrapings to detect OC. While one study identified a panel of three genes (*AMPD3*, *NRN1*, and *TBX15*) with a sensitivity and specificity of 81% and 84%, respectively [136], another study identified *POU4F3* and *MAGI2* with a sensitivity and specificity of 61% and 62–69%, respectively, as promising biomarkers for OC detection [137]. A feasibility study on quantitative methylation-specific PCR on cervical scrapings identified 67% of patients with cervical cancer using a panel of genes [138]. Notably, the WID-OC index, a DNA methylation signature in cervical cells, has proven capable of detecting OC (AUC: 0.76) and endometrial cancer (AUC: 0.81) [139]. These studies suggest that DNA methylation testing holds promise as a non-invasive method for OC detection and risk assessment. However, the limited number of studies on this approach challenge its applicability.

## 7. Challenges in Early Diagnosis

### 7.1. Biological Heterogeneity of OC

OC is characterized by inter-subtype and intra-tumoral heterogeneity, challenging early diagnosis and effective treatment [140,141]. This heterogeneity at genomic, epigenomic, and proteomic levels contributes to treatment resistance and tumor recurrence [142]. Intra-tumoral heterogeneity arises from clonal evolution and microenvironmental influences on cancer stem cells [142]. Advanced technologies such as NGS, mass spectrometry, and protein array analysis have furthered our understanding of the molecular complexity of OC [112]. However, early detection remains challenging, with most patients being diagnosed at advanced stages [112]. Research on leveraging tumor heterogeneity to develop personalized therapies and improve patient outcomes is the current need [142]. Understanding the protein-level translation of genomic and epigenomic heterogeneity may help improve survival outcomes in patients with OC [112].

### 7.2. Socioeconomic Barriers to Early Screening and Diagnosis

Socioeconomic status significantly impacts OC outcomes; notably, increased poverty is associated with an advanced-stage diagnosis [143]. Multiple barriers, including hospital and physician volumes, geographic distance from care facilities, and demographic factors, hinder access to care [144]. Patients from more impoverished areas experience longer diagnostic and treatment intervals, and they are less likely to receive surgery and chemotherapy [145]. Racial and ethnic disparities in OC survival rates are attributed to a combination of genomic, socioeconomic, and cultural factors. Language barriers, transportation limitations, and high comorbidity rates in certain populations further contribute to disparities [146]. Guideline-adherent care is associated with patient proximity to high-volume hospitals, white race, and high socioeconomic status [144]. Addressing socioeconomic barriers and reducing healthcare disparities are key policy targets to improve OC outcomes across diverse populations.

### 7.3. Current Knowledge and Research Gaps

In the absence of a standardized method in current guidelines, early diagnosis and screening for OC remain significant challenges [147]. Among >200 proposed tumor markers, only CA-125 and HE4 have been clinically tested [147]. Research gaps exist across the continuum of OC care, from prevention to end-of-life care [148]. Current epidemiological studies are limited by outdated exposure information and the need for larger collaborative efforts, which help achieve meaningful sample sizes for histotype-specific analyses [56]. Identification of novel modifiable risk factors and the development of better risk prediction models are crucial [56]. Future research should focus on biomarkers, multiplex panels, and multimodal algorithms combining tumor markers, cfDNA, and ultrasound [147]. However, convincing data on mortality reduction from randomized controlled trials remain lacking [147,149].

## 8. Future Directions

### 8.1. Increase Awareness

Awareness of OC symptoms and risk factors is generally low among women, with only 40% reporting familiarity with symptoms [150]. Moreover, knowledge gaps exist among healthcare providers, highlighting the need for continued education [150]. Educational interventions such as the Inside Knowledge campaign help in increasing awareness and facilitate confident discussions on gynecological cancers [151]. Targeted health education sessions can significantly improve knowledge about OC among working women [152]. However, misconceptions persist, such as believing cervical smears screen for OC or that oral contraceptives increase risk [153]. Most women recognize that early detection via screening could reduce mortality; nevertheless, a clear need for improved public understanding of OC risks and symptoms exists [153]. These findings underscore the importance of continued efforts to increase awareness among both patients and healthcare providers.

### 8.2. Integration of Multiomics Approaches for Precision Diagnostics

Multiomics is emerging as a powerful tool for exploring molecular mechanisms and identifying biomarkers in OC. Integrated data from genomics, transcriptomics, proteomics, and metabolomics provide comprehensive insights into cancer development and progression [154]. Machine learning algorithms, particularly deep learning techniques such as variational autoencoders, have helped analyze high-dimensional multiomics data, addressing challenges of data imbalance and dimensionality reduction [155]. Integrated multiomics reportedly outperform single-omics in identifying diagnostic and prognostic biomarkers, as well as potential therapeutic targets [156]. For example, combined metabolomics and proteomics analysis has revealed novel biomarkers and signaling pathways in HGSOC [157]. Advancements in integrated multiomics and machine learning applications herald more precise diagnostic and personalized treatment strategies in OC.

### 8.3. Development of Cost-Effective and Scalable Screening Methods

The urgent need for improved OC screening methods, particularly for early-stage detection, continues to be highlighted. Current diagnostic tools such as TVUS and CA-125 lack sufficient sensitivity and specificity [158]. Potential biomarkers under investigation include autoantibodies, ctDNA, and blood-based DNA methylation [159]. Liquid biopsies, which analyze biomarkers in blood, urine, and uterine lavage samples, are a novel diagnostic strategy [55,158]. Other promising strategies include two-stage screening, combining CA-125 tracking with ultrasound [159], and integrated microRNA profiling, which has demonstrated a high accuracy in differentiating OCs from other diseases [160]. Advanced imaging techniques such as magnetic relaxometry and autofluorescence may improve detection sensitivity [159]. These methods aim to improve early diagnosis, and ultimately, reduce mortality rates in patients with OC [55,159].

### 8.4. Collaborative Efforts

Collaborative efforts in global health initiatives for OC are part of a broader focus on addressing cancer disparities in low- and middle-income countries (LMICs). Although these countries bear 60% of the global cancer burden, their global cancer spending accounts for only 5% [161,162]. Key challenges include limited resources for screening, advanced-stage diagnoses, and inadequate treatment options [163]. Accordingly, international organizations such as the American Society of Clinical Oncology, Union for International Cancer Control, and WHO have launched initiatives focusing on prevention, early detection, and resource-adapted interventions [161]. The Breast Health Global Initiative’s resource-stratified guideline has been adopted by oncology societies to improve care in resource-limited settings [161]. Collaboration among various stakeholders, including the pharmaceutical industry, health authorities, and non-profit organizations, is crucial for improving OC outcomes in LMICs [161,162].

## 9. Conclusions

Early diagnosis of OC remains a significant challenge owing to its asymptomatic nature and lack of effective screening methods. Current diagnostic tools, including biomarkers CA-125 and HE4, have varied sensitivity and specificity, particularly in early stages. Genomics and proteomics-based research have identified several potential biomarkers, including gene- and protein-based biomarkers, and emerging indicators such as microRNA and metabolites. Novel approaches such as the ROCA and multivariate index assays (OVA1 and ROMA) can potentially enhance diagnostic accuracy. Imaging techniques, including ultrasound, MRI, and PET/CT, crucially complement biomarker testing. However, the need for biomarkers with both high specificity and sensitivity for early diagnosis remains. Further validation and clinical trials are required before implementing new biomarker tests in routine clinical practice.

## Figures and Tables

**Table 1:** Search strategy outline.

Items	Specifications
Time frame	From inception to 24 December 2024
Database	PubMed, Scopus, Web of Science, and Embase
Search terms	“Ovarian cancer” and “diagnosis”
Inclusion criteria	SCI-indexed research articles written in English
Selection process	Two independent reviewers evaluated the titles and abstracts for eligibility

SCI, Science Citation Index.

**Table 2:** Sensitivity and specificity of various imaging modalities.

Imaging Modality	Sensitivity (%)	Specificity (%)	PPV (%)	NPV (%)	Comments
TVUS [58]	84.9	98.2	5.3	Not specified	High sensitivity and specificity, albeit low PPV, leading to unnecessary surgeries for benign masses
CT [63]	40.7–92.1	57.1–89.1	80	58.3	Effective for radiomics and sarcopenia evaluation, promising in distinguishing benign from malignant tumors
MRI [71]	86.7	81.9	76.7	84.6	Diffusion-weighted imaging and MR perfusion are useful in the diagnosis of ovarian tumors
PET/CT [77]	26.7 (lymph node), 41.7–67.8 (peritoneal carcinomatosis)	90.9 (lymph node), high for staging	100	42.9	Revolutionizes staging and treatment, with high specificity albeit variable sensitivity for different sites

TVUS: transvaginal ultrasound, CT: computed tomography, MRI: magnetic resonance imaging, PET: positron emission tomography, PPV: positive predictive value, NPV: negative predictive value.

**Table 3:** Diagnostic performance of biomarkers and combined modalities in ovarian cancer detection.

Markers	Sensitivity (%)	Specificity (%)	PPV (%)	NPV (%)
CA-125 [88]	80.1	53.6	48.4	83
CA-125 + MI [85]	98.1	80.8	40.9	99.7
ROCA, CA-125 [87]		92	4.6	
ROCA, CA-125 + TVUS [89]		99.9	40	
CA 125 + symptoms [90]	89.3	83.5		
CA-125 + HE4 + symptoms [93]	79	91	83	89

MI: morphology index; ROCA: Risk of Ovarian Cancer Algorithm; TVUS: transvaginal ultrasound, PPV: positive predictive value, NPV: negative predictive value.

## Data Availability

No new data were created or analyzed in this study. Data sharing is not applicable to this article.

## Abbreviations

The following abbreviations are used in this manuscript:

OC	Ovarian cancer
AI	Artificial intelligence
CT	Computed tomography
MRI	Magnetic resonance imaging
PET	Positron emission tomography
HE4	Human epididymis protein 4
TVUS	Transvaginal ultrasound
FDG	[^18^F]Fluorodeoxyglucose
ROMA	Risk of Ovarian Malignancy Algorithm
ROCA	Risk of Ovarian Cancer Algorithm
WHO	World Health Organization
LMICs	Low- and middle-income countries
HGSC	High-grade serous carcinoma
HGSOC	High-grade serous ovarian carcinoma
LGSC	Low-grade serous carcinoma
CCCO	Clear cell carcinoma of the ovary
MOC	Mucinous ovarian carcinoma
AUC	Area under the curve
GCT	Germ cell tumor
SCST	Sex cord/stromal tumor
PPV	Positive predictive value
NPV	Negative predictive value
STIC	Serous tubal intraepithelial carcinoma
EOC	Endometrioid ovarian carcinoma
O-RADS	Ovarian-Adnexal Reporting and Data System
PCI	Peritoneal carcinomatosis index
ctDNA	Circulating tumor DNA
CTCs	Circulating tumor cells
NGS	Next-generation sequencing
cfDNA	Cell-free DNA
CICs	Cortical inclusion cysts
OSE	Ovarian surface epithelium
